# Supplementary material for: Association of IL-17A and IL-10 Polymorphisms with Juvenile Idiopathic Arthritis in Finnish Children
Source: Int J Mol Sci. 2024 Jul 30;25(15):8323. doi: 10.3390/ijms25158323 (PMC11311899; doi:10.3390/ijms25158323)
Supplement: Supplementary file 1 [file ijms-25-08323-s001.zip › ijms-3037383-supplementary.pdf]

Supplementary Table S1:

SNPs	Genome 1000, n= 99 (%)	Oligoarthritis, n=51 (%)		Polyarthritis, n=34 (%)	OR (95% Cl), P-value[3]	OR (95% Cl), P-value[4]	OR (95% Cl), P-value[5]	OR (95% Cl), P-value[6]	OR (95% Cl), P-value[7]
IL-17A		Persistent (n=44)	Extended (n=7)						
rs9395767									
AA	31 (31.3)	15 (34.1)	3 (42.9)	7 (20.6)					
AT	43 (43.4)	19 (43.2)	1 (14.3)	16 (47.1)	0.305	0.375	0.238	0.926	0.305
TT	25 (25.2)	10 (22.7)	3 (42.9)	11 (32.4)					
AT + TT[2]	68 (68.7)	29 (65.9)	4 (57.1)	27 (79.4)	0.690 (0.14 – 3.49), 0.652	2.00 (0.71 – 5.64), 0.189	2.89 (0.52 – 16.03), 0.670	0.923 (0.44 – 1.96), 0.835	0.637 (0.13 – 3.02), 0.567
A-allele	105 (53.0)	56 (54.9)	7 (50.0)	30 (44.1)					1.13 (0.38 – 3.34),
T-allele	93 (47.0)	46 (45.1)	7 (50.0)	38 (55.9)	1.21 (0.40 – 3.72), 0.345	1.54 (0.83 – 2.86), 0.169	1.27 (0.40 – 4.01), 0.688	0.93 (0.57 – 1.50), 0.758	0.826
rs4711998									
GG	47 (47.5)	20 (45.5)	2 (28.6)	13 (38.2)					
AG	45 (45.5)	18 (40.9)	5 (71.4)	18 (52.9)	0.272	0.543	0.571	0.446	0.380
AA	7 (7.1)	6 (13.6)	0 (0.0)	3 (8.8)					
AG + AA[2]	52 (52.5)	24 (54.5)	5 (71.4)	21 (61.8)	2.08 (0.36 – 11.92), 0.402	1.46 (0.66 – 3.24), 0.426	0.65 (0.11 – 3.83), 0.629	1.09 (0.53 – 2.21), 0.858	2.26 (0.42 – 12.20), 0.332
G-allele	139 (70.2)	67 (65.7)	9 (64.3)	44 (64.7)	1.06 (0.33 – 3.42),	1.04 (0.55 – 1.99),	0.98 (0.30 – 3.26),	1.23 (0.74 – 2.05),	
A-allele	59 (29.8)	35 (34.3)	5 (35.7)	24 (35.3)	0.103	0.900	0.976	0.425	1.31 (0.42 – 4.07),
rs8193036									0.642
TT	40 (40.4)	15 (34.1)	0 (0.0)	10 (29.4)					
CT	40 (40.4)	18 (40.9)	6 (85.7)	17 (50.0)	0.072	0.724	0.174	0.383	**0.046***
CC	19 (16.2)	11 (25.0)	1 (14.3)	7 (20.6)					
CT + CC[2]	59 (59.6)	29 (65.9)	7 (100.0)	24 (70.6)	1.24 1.06 – 1.46), 0.066	1.24 (0.47 – 3.26), 0.661	0.89 (0.82– 0.97), 0.099	1.49 (0.71 – 3.11), 0.294	0.89 (0.82 – 0.970), **0.024***
T-allele	126 (63.6)	54 (52.9)	6 (42.9)	37 (54.4)	1.50 (0.49 – 4.63),	0.94 (0.51 – 1.74),	0.628 (0.197 – 2.07),	1.44 (0.89 – 2.32),	2.15 (0.72 – 6.44),
C-allele	78 (36.4)	48 (47.1)	8 (57.1)	31 (45.6)	0.481	0.851	0.433	0.140	0.170
rs2275913									
GG	33 (33.3)	9 (20.5)	0 (0.0)	8 (23.5)					
GA	47 (47.5)	22 (50.0)	7 (100.0)	14 (41.2)	**0.046***	0.739	**0.018***	0.200	**0.027***
AA	19 (19.2)	13 (29.5)	0 (0.0)	12 (35.3)					
GA + AA[2]	66 (66.6)	35 (79.5)	7 (100.0)	26 (76.5)	1.2 (1.05 – 1.37), 0.187	0.84 (0.24 – 2.46), 0.744	1.30 (1.06 – 1.52), 0.153	1.94 (0.84 – 4.52), 0.119	0.904 (0.84 – 0.97), 0.066
G-allele	113 (57.0)	47 (46.1)	7 (50.0)	30 (44.1)	0.855 (0.280 – 2.61),	1.08 (0.58 – 2.01),	1.27 (0.40 – 4.01),	1.56 (0.96 – 2.52),	1.33 (0.45 – 3.93),
A-allele	85 (43.0)	55 (53.9)	7 (50.0)	38 (55.9)	0.782	0.252	0.688	0.071	0.607
IL-10									
**rs1800896**									
TT	36 (36.4)	10 (22.7)	1 (14.3)	8 (23.5)					
TC	47 (47.5)	25 (56.8)	5 (71.4)	20 (58.8)	0.765	0.953	0.812	0.373	0.490
CC	16 (16.2)	9 (20.5)	1 (14.3)	6 (17.6)					
TC + CC[2]	63 (63.6)	34 (77.3)	6 (85.7)	26 (76.5)	1.77 (0.19 – 16.43), 0.614	0.96 (0.33 – 2.76), 0.934	0.54 (0.06 – 5.19), 0.591	1.78 (0.79 – 4.03), 0.165	3.12 (0.36 – 27.14), 0.28
T-allele	119 (60.1)	52 (51.0)	7 (50.0)	36 (52.9)	1.04 (0.34 – 3.18),	0.92 (0.50 – 1.71),	0.89 (0.28 – 2.81),	1.45 (0.90 – 2.34),	1.51 (0.51 – 4.46),
C-allele	79 (39.9)	50 (49.0)	7 (50.0)	32 (47.1)	0.955	0.802	0.841	0.131	0.460
**rs1800871**									
GG	59 (59.6)	26 (59.1)	4 (57.1)	19 (55.9)					
GA	33 (33.3)	18 (40.9)	3 (42.9)	14 (41.2)	0.999	0.515	0.900	0.165	0.713
AA	7 (7.1)	0 (0.0)	0 (0.0)	1 (2.9)					
GA + AA[2]	40 (40.4)	18 (40.9)	3 (42.9)	15 (44.1)	1.08 (0.22 – 5.44), 0.999	1.14 (0.46 – 2.82), 0.776	1.05 (0.204 – 5.44), 0.951	1.02 (0.50 – 2.10), 0.955	1.11 (0.24 – 5.21), 0.898
G-allele	151 (76.3)	81 (79.4)	11 (78.6)	52 (76.5)	1.05 (0.27 – 4.11),	1.19 (0.57 – 2.48),	1.13 (0.28 – 4.55),	0.83 (0.47 – 1.49),	0.88 (0.23 – 3.27),
A-allele	47 (23.7)	21 (20.6)	3 (21.4)	16 (23.5)	0.942	0.649	0.865	0.537	0.844

Data is presented as numbers (n) of children and valid percentages (%).[1]Genome 1000 FIN population. The odds ratio (OR) and the 95 % confidence intervals (95% CI) were calculated. [2]Dominant model where wild-type was compared with heterozygous and homozygote variant. Fisher's exact tests were used to analyse P- values and P-values < 0.05 were considered significant (*).P-value and OR when analyses were done between [3] Persistent- and Extended Oligo arthritis, [4] Persistent Oligo arthritis and Poly arthritis, [5] Extended oligo arthritis and Poly arthritis, [6] Persistent oligo arthritis and controls, [7] Extended oligo arthritis and controls.
